# Nanobody-Based Lateral Flow Assay for Rapid Zika Virus Detection

**DOI:** 10.1021/acssynbio.4c00819

**Published:** 2025-03-07

**Authors:** Yuli Peng, Atheer Alqatari, Fabian Kiessling, Dominik Renn, Raik Grünberg, Stefan T. Arold, Magnus Rueping

**Affiliations:** † KAUST Catalysis Center (KCC), Division of Physical Sciences and Engineering, King Abdullah University of Science and Technology (KAUST), Thuwal 23955, Kingdom of Saudi Arabia; ‡ KAUST Center of Excellence for Smart Health, Biological and Environmental Science and Engineering Division, King Abdullah University of Science and Technology (KAUST), Thuwal 23955-6900, Kingdom of Saudi Arabia; § Institute for Experimental Molecular Imaging (ExMI), University Hospital, RWTH Aachen University, Forckenbeckstraße 55, Aachen D-52074, Germany

**Keywords:** lateral flow immunoassay, VHH, nanobody, Zika, viral antigen detection

## Abstract

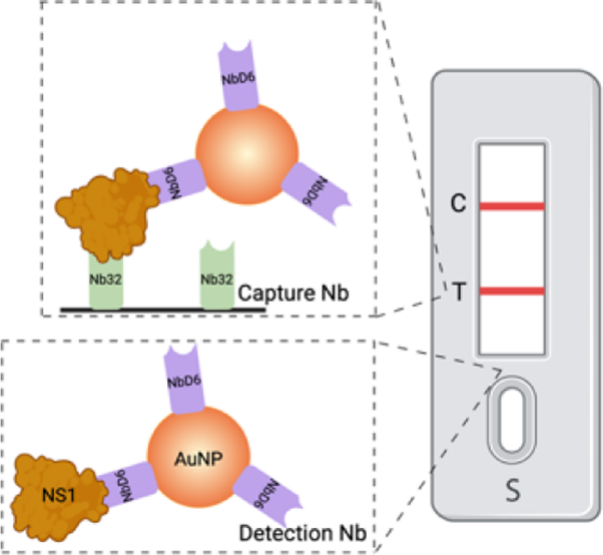

Zika virus infections remain severely underdiagnosed due to their initial mild clinical symptoms. However, recent outbreaks have revealed neurological complications in adults and severe deformities in newborns, emphasizing the critical need for accurate diagnosis. Lateral flow assays (LFAs) provide a rapid, cost-effective, and user-friendly method for antigen testing at point-of-care, bedside, or in home settings. LFAs utilizing nanobodies have multiple benefits over traditional antibody-based techniques, as nanobodies are much smaller, more stable, and simpler to manufacture. We introduce a nanobody-based LFA for the rapid identification of Zika virus antigens. Starting from two previously reported nanobodies recognizing the Zika nonstructural protein 1 (NS1), we evaluate periplasmic and cytosolic nanobody expression and test different purification tags and immobilization strategies. We quantify nanobody binding kinetics and validate their mutually noncompetitive binding. Avidity effects boost the capture of the tetrameric target protein by 3 orders of magnitude and point to a general strategy for higher sensitivity LFA sensing. The nanobody LFA detects Zika NS1 with a limit of detection ranging from 25 ng/mL in buffer to 1 ng/mL in urine. This nanobody-LFA has the potential to facilitate on-site and self-diagnosis, improve our understanding of Zika infection prevalence, and support public health initiatives in regions affected by Zika virus outbreaks.

## Introduction

The Zika virus (ZIKV) belongs to the Flaviviridae family, which also includes Dengue, West Nile virus, Japanese encephalitis, and yellow fever. These viruses are primarily transmitted by ticks and mosquitoes and can cause similar symptoms, but each requires distinct treatments.^1,2^ ZIKV infections often proceed asymptomatically or with mild symptoms. However, the virus can be transmitted through blood transfusions and vertically during pregnancy.^1^ Studies have linked *in-utero* ZIKV infections with microcephaly, a condition characterized by an abnormally small head in newborns, leading to developmental and neurological impairments. ZIKV infections can also affect the nervous system or cause Guillain-Barré Syndrome in adults.^3,4^ In 2015, Brazil and several South American countries experienced a ZIKV epidemic which was later declared a global public health emergency by the World Health Organization (WHO).^5^ To date, around 39 regions in North and South America continue to report consistent ZIKV transmission.^3^ Additionally, in 2018, India reported a resurgence of infections.^1^ The often asymptomatic representation complicates clinical surveillance and Zika remains underdiagnosed.^6,7^ For example, the mosquito vector, *Aedes aegypti*, is endemic to Saudi Arabia but no cases have been reported to date. However, in a study on 410 pregnant local women, all were negative for acute virus but 6% were positive for ZIKV IgM, suggesting previous infection.^7^ The substantial underdiagnosis of ZIKV infections may lead to undiagnosed health complications in newborns.^6,7^

ZIKV infection is characterized by two distinct stages: the acute phase (within the first 5 days of infection) and the convalescence phase (extending 6 days after the acute phase).^8,9^ During the acute phase, the virus genome and antigens can be detected in various body fluids, including blood, serum, urine, saliva, semen, and amniotic fluid.^10,11^ Among these, semen has the highest virus load and the longest virus persistence, but serum and urine are most commonly used for virus detection.^12−14^ In the convalescence phase, antibodies produced by the immune response, immunoglobulin M (IgM) and IgG, can be detected.^9^

Several approaches have been developed to detect the ZIKV genome or viral antigens.^9^ Nucleic acid amplification methods such as reverse-transcriptase polymerase chain reaction (RT-PCR), loop-mediated isothermal amplification (LAMP), and recombinase polymerase amplification (RPA) are used for genome detection.^15−17^ These methods have very high sensitivity but require relatively complex equipment and rely on nucleic acid extraction and sample processing. Alternatively, enzyme-linked immunosorbent assay (ELISA) is widely used for the highly sensitive detection of Zika nonstructural protein 1 (NS1), or anti-Zika IgM, or IgG.^18−20^ However, ELISA requires specialized instruments and trained personnel, limiting its use to centralized laboratories.^21^ Consequently, neither nucleic acid assays nor protein detection via ELISA is suitable for self-testing. Moreover, the commonly used ELISA for detecting patient antibodies has significant limitations: it often fails to identify the virus during its critical early phase and is prone to cross-reactivity with other homologous flaviviruses, such as Dengue virus.^1,22^ As of 2024, the U.S. Food and Drug Administration (FDA) has authorized four ELISA ZIKV IgM diagnostic tests as well as several RNA-based tests for emergency use.^9,23^ Acknowledging the limitations of these methods, the FDA actively encourages the development of alternative commercial diagnostics.^23^ This highlights the ongoing need for effective and accessible point-of-care diagnostics for ZIKV.

Lateral flow assays (LFAs) are a well-established platform for the rapid detection of molecular antigens in nearly any setting, including for self-testing at home.^24^ While many laboratory-based techniques are more sensitive or quantitative, LFAs outcompete most other methods in terms of simplicity, speed, and cost. Consequently, LFAs became the first line of diagnostic defense during the severe acute respiratory syndrome coronavirus 2 (SARS-CoV-2) pandemic.^25,26^ In the context of a disease outbreak, ready access to an appropriate LFA rapid test greatly facilitates containment even under adverse conditions.^24,27^

Several antibody-based LFAs for the detection of ZIKV antigen or patient antibodies have been reported in the literature but generally require advanced technical setups for their use or readout. Sanchez-Purra and colleagues developed a Raman spectroscopy-based LFA.^28^ Xu and colleagues described a high-sensitivity LFA using fluorescent carbon dots-based silica colloids conjugated with antibodies relying on a special ultraviolet excitation.^29^ Likewise, a portable smartphone-based fluorescent LFA proposed by Zhen and colleagues requires an ultraviolet lamp.^30^

By contrast, conventional gold nanoparticle (AuNP)-based LFAs are rapid, easy to perform and can be read out by naked eye.^31,32^ However, the production of disease-specific antibody protein from mammalian cell culture represents a major bottleneck and cost factor in their production. Recently, LFAs have been developed in which complex regular antibodies are replaced by single-domain antibodies, known as nanobodies.^33−35^ Nanobodies are the antigen-recognizing domains derived from a class of single-chain antibodies found in camelids, with llamas and camels being the most common sources. Compared to conventional multichain, multidomain antibodies, nanobodies are ten times smaller, much more stable, and can bind their targets with equal or superior affinity.^36^ Unlike regular antibodies, nanobodies can be produced more quickly, inexpensively, and in larger quantities from *Escherichia coli* (*E. coli*) bacterial cultures. Triana and colleagues identified nanobodies specifically targeting ZIKV NS1 and reported negligible cross-reactivity with other flavivirus NS1 proteins. They used these nanobodies for detecting ZIKV NS1 in serum samples by ELISA.^4,37^ NS1 is a critical viral protein that has emerged as an important diagnostic biomarker for multiple flavivirus infections.^38^ This multifunctional protein exists in two distinct forms: a membrane-associated form that is essential for viral replication and a soluble form that is actively secreted into the bloodstream.^38^ The secreted NS1 can be readily detected in various noninvasive biological specimens, including blood serum, urine, and saliva. The protein begins to appear in patient samples within hours of initial viral infection, significantly earlier than detectable antibody responses. This early secretion pattern, combined with its presence in easily accessible body fluids, makes NS1 an ideal target for rapid diagnostic testing.^39^ We here further characterize anti-NS1 nanobodies and integrate them into a nanobody-based lateral flow immunoassay for rapid and efficient POC diagnostics of ZIKV during the acute infection phase.

## Material and Methods

### Materials

All target proteins were produced by commercial providers in HEK293 cells with C-terminal His-tag. The ZIKV SPH2015 NS1 target protein was purchased from Sinobiological (cat: 40544-V07H, Figure S7). All four of the Dengue NS1 target proteins were purchased from the Native Antigen Company (cat: DENVX4-NS1–100). Anti-VHH antibody (cat: 128-005-232) was purchased from Jackson ImmunoResearch (West Grove, PA, USA). All the components used in LFA strips (Product code: 07.700.30) were purchased from ClaremontBio (Upland, CA, USA). Gold nanoparticles (cat: AUNR40, AUXR40) were purchased from nanoComposix (San Diego, CA, USA). All other chemicals and solvents in case not specified were purchased from Sigma-Aldrich (St. Louis, MO, USA) and ThermoFisher Scientific (Waltham, MA, USA).

### Expression, and Purification of Strep-Tagged Nanobodies

The nanobody constructs were designed with a C-terminal StrepTag-II in vector pJE411c (in-house modified from DNA 2.0 pJExpress411, Figure S6), codon optimized for *E. coli* expression using an in-house Python script based on DNAChisel. The constructs were gene-synthesized by Twist Bioscience.^40,41^

Plasmids were transformed by heat shock into chemically competent *E. coli* BL21(DE3) cells. Overnight starter cultures were inoculated from a single colony and used to inoculate 1L 2xYT production cultures at 1:100 ratio, supplemented with 50 mg/L kanamycin and 0.5% w/v glucose. Cultures were incubated at 37 °C and 220–250 rpm, induced with isopropyl beta-D-1-thiogalactopyranoside (IPTG, 0.25 mM final concentration) between 0.6 to 0.8 OD_600_ and expressed for 4 h at 37 °C and 220–250 rpm. Cells were harvested by centrifugation for 10 min at 6000*g*, washed twice with 20–30 mL ice-cold PBS and stored at −80 °C.

Proteins were extracted from the periplasm using osmotic shock.^42^ The pellet was thawed and resuspended in extraction buffer I (30 mM Tris-HCl pH 8.0, 20% w/v sucrose, 1 mM EDTA). Resuspended cells were stirred slowly at room temperature for 10 min until the solution turned homogeneous. Supernatant I was collected by centrifugation (10 min @ 10,000*g*, 4 °C). The pellet was resuspended in 30 mL ice-cold 5 mM Mg_2_SO_4_ and then stirred for 15 min on ice and supernatant II was again collected by centrifugation. Both supernatants were combined, centrifuged at 30,000*g* for 45 min at 4 °C and filtered through MiraCloth filter (Merck Millipore, Cat: 475855–1R). The clarified sample was applied to a 5 mL StrepXT column (Cytivia) washed with binding buffer (100 mM Tris-HCl pH 8.0, 150 mM NaCl, 1 mM EDTA, 10% glycerol) and eluted after 20 min incubation with 50 mM D-biotin in binding buffer. The column was regenerated between purifications with 50 mM NaOH. Fractions were verified by SDS-PAGE, pooled and concentrated with 10 kDa centrifugation filters (Amicon Ultra, Merck Millipore, Cat: UFC901024) and subjected to gel filtration on a 16/600 Superdex 75 size exclusion column (Cytivia) in gel filtration buffer (20 mM HEPES pH 7.4, 300 mM NaCl, 1 mM EDTA, 10% glycerol). Fractions were analyzed, pooled, concentrated and final concentrations determined by 280 nm absorbance (NanoDropOne, ThermoFisher) using the sequence-specific extinction coefficient. Samples were aliquoted, flash frozen in liquid N_2_ and stored at −80 °C in gel filtration buffer.

### Expression, and Purification of GST-Tagged Nanobodies

The nanobody constructs were designed with an N-terminal GST-tag (Figure S6) in vector pET29b, codon optimized for *E. coli* expression and synthesized by Twist Bioscience. Plasmids were transformed into *E. coli* SHuffle T7 competent cells (New England Biolabs, C3026J) using heat shock according to the manufacturer. Overnight starter cultures were inoculated from a single colony and used to inoculate 1 L LB (Lennox) cultures at 1:100 ratio, supplemented with 50 μg/L kanamycin. Cultures were incubated at 30 °C and 200 rpm, induced with 0.5 mM IPTG between 0.4 to 0.6 OD_600_ and expressed for 36 h at 16 °C. Cells were harvested by centrifugation at 6000 g at 4 °C for 30 min, resuspended in lysis buffer [1x phosphate-buffered saline (PBS), 10 mM MgCl_2_, 10 mM Imidazole, 10 μg/mL DNase (SIGMA DN25), 1 tablet protease inhibitor (ThermoScientific Cat: A32963) per 50 mL lysis buffer] and lysed on a Cell Disruptor (Constant Systems, CF1). Cell debris was removed by centrifugation at 24000*g* at 4 °C for 1 h.

GST-tagged nanobodies were purified using Glutathione MagBeads (GenScript L00895) following the procedures provided in the product manual. After a 2 h incubation with MagBeads at 4 °C, nanobodies were eluted with 10 mM reduced glutathione in 50 mM Tris-HCl, pH 8.0. Proteins were further purified by size exclusion chromatography as described above albeit using PBS buffer. Purified proteins were stored at −80 °C in PBS buffer supplemented with 20% glycerol.

### BLI Binding Kinetics and Competitive Assay

Biolayer interferometry (BLI) binding kinetics was measured on an Octet RH96 (Sartorius), following the measurement protocol shown in Table 1, and analyzed in the Octet Analysis software version 13.0.0.32. Sensors tips were hydrated for 10 min in binding buffer (PBS, with 0.02% Tween-20 and 0.1% bovine serum albumin). Experiments were performed at 25 °C and 1000 rpm shaking in 96 and 384 well plates in 200 and 80 μL volumes, respectively. Optimized loading conditions for NS1 glycoprotein onto Ni-NTA sensor tips were 600 s, using a protein concentration of 100 nM, in binding buffer. Optimized conditions for loading GST-tagged nanobodies onto anti-GST sensor tips were 400 s, with a protein concentration of 100 nM, in binding buffer. Sensor tips were regenerated (up to five times) with 10 mM Glycine pH 2.0, neutralized in binding buffer and activated with 10 mM NiSO_4_.

**Table 1:** Summary of the Steps Used for the Octet Experiments

Step	Step name	Time [s]	Solution
1	Baseline	60	Buffer
2	Loading	400–600	100 nM ligand protein
3	Wash	30	Buffer
4	Baseline 2	120	Buffer
5	Association	450	Variable analyte conc.
6	Dissociation	600	Buffer
7	Regeneration/Neutralization	60	10 mM Glycine pH 2.0
8	Activation	30	10 mM NiSO_4_

For the competitive assay, Ni-NTA tips loaded with NS1 were first saturated with ZIKV_Nb32 and a regular binding experiment was then performed with different concentrations of ZIKV_NbD6 as the competing nanobody.

### Characterization of Zika NS1 by Mass Photometry

The molecular mass distribution of the Zika NS1 glycoprotein was measured on the Refeyn TwoMP Mass Photometer using the AcquireMP software (v2024 R2.1) following the manufacturer’s instructions. Per measurement, 5 μL of PBS buffer was mixed with 5 μL of sample in the 10 μL sample well and collision events were recorded for 60 s. Zika NS1 glycoprotein was tested at four different final concentrations: 25, 50, 100, and 250 nM. Commercially purchased proteins—ovalbumin (Cytiva, Gel Filtration Calibration Kit), bovine serum albumin (BSA, Sigma-Aldrich, 9048-46-8), and catalase (Sigma-Aldrich, C100–50MG)—were used for mass calibration at concentrations of 50 nM, 50 nM, and 17 nM, respectively. Data were analyzed in the manufacturer’s DiscoverMP software (v2024 R2.1).

### Preparation of Nanobody-AuNP Biconjugate

Nanobodies were dialyzed overnight against 8 mM potassium phosphate buffer (pH 7.4) and conjugated with Bioready 40 nm Gold Nanospheres-Carboxyl nanoparticles (OD 20, nanoComposix) according to the manufacturer’s protocol with the following adjustments: Sulfo-NHS and 1-ethyl-3-(3-(dimethylamino)propyl) carbodiimide-HCl (EDC) were made freshly at 10 mg/mL in water before addition to the carboxyl AuNP solution. After 30 min incubation with 20 rpm rotation on a Tube Revolver Rotator (Thermo Scientific) at room temperature, excess sulfo-NHS and EDC were washed by centrifugation at 3800 g for 10 min. The supernatant was removed, and the pellet was resuspended in reaction buffer (5 mM potassium phosphate, pH 7.4, 0.5% PEG 12000). This wash step was repeated once. Then 30 μg nanobody per mL AuNP was added for conjugation. After rotation at room temperature for 1 h, the reaction was quenched with 50% (w/v) hydroxylamine and incubated for another 10 min. The reaction mix was centrifuged at 3800 g for 10 min, the supernatant was removed and the pellet was resuspended in reaction buffer (5 mM potassium phosphate, pH 7.4, 0.5% 12000 MW PEG). This wash step was repeated twice. The washed pellet was resuspended in storage buffer (DDS Diagnostic Company, Catalog 1933) and stored at 4 °C.

### Preparation of LFA Strips

The conjugate pad (Glass fiber Ahlstrom 8964) was pretreated with conjugate blocking buffer (10 mM phosphate buffer, pH 7.4, 2% (w/v) BSA, 2.5% (w/v) sucrose, 0.3% (w/v) PVP, 1%(w/v) Triton X-100, and 0.02% (w/v) NaN_3_) and dried at 37 °C for 1 h. The nanobody-AuNP biconjugate was applied using the automated lateral flow reagent dispenser (ALFRD, ClaremontBio) at 20 μL/cm. The detection pad used in this assay was a nitrocellulose membrane (MD100) and the proteins for control line (0.6 mg/mL) and test lines (1.5 mg/mL) were dispensed on it at 0.83 μL/cm. The nitrocellulose membranes were dried at 37 °C for 30 min after printing and then blocked with blocking buffer (5 mM potassium phosphate, pH 8.0, 0.6% BSA) for 30 min and dried at 37 °C for another 30 min. For assembly, sample pad (glass fiber Ahlstrom 8964), conjugate pad, nitrocellulose membrane and absorbent pad (AHL238 cotton fiber) were pasted on the backing plate (ClaremontBio Adhesive Backing Card) and cut into 3 mm strips using the High-Speed Test Strip Guillotine Cutter (Werfen Equipment). Freshly made LFA strips were stored at 4 °C and room temperature with desiccant packs.

### Detection of Antigens in Buffer or Spiked Body Fluids

Standard samples were prepared by diluting antigen with running buffer (20 mM Tris/HCl, pH 8.0, 0.5% BSA, 0.5% Triton X-100). ZIKV NS1 protein (strain: SPH2015) spiked into bovine urine (ERM-BB386) or Normal Human Serum (Sigma-Aldrich) were used to simulate actual biological samples. 50 μL of each sample was added to the sample pad of LFA strips.

## Results and Discussion

### Experimental Design

**Figure 1:**
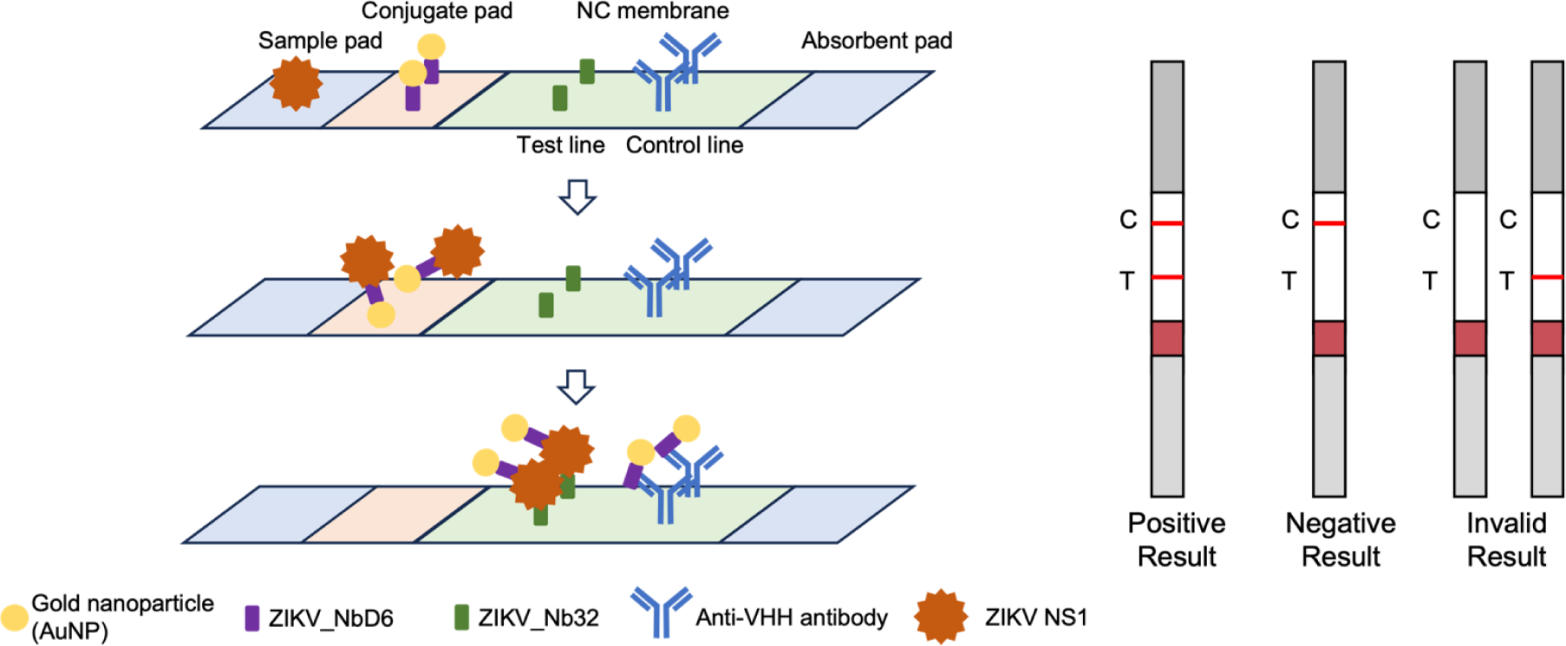
Scheme of nanobody-based sandwich lateral flow. When the ZIKV NS1 protein antigen is added, it initially forms a complex with the AuNP-conjugated ZIKV_NbD6. This antigen-nanobody-AuNP complex is then immobilized on the test line by the capture nanobody ZIKV_Nb32. Any remaining nanobody-AuNP complexes are subsequently immobilized on the control line by an anti-VHH antibody. The LFA is considered positive when both the test and control lines are visible to the naked eye. It is negative when only the control line is visible. The assay is invalid if the control line is not visible.

Our overall LFA setup is shown in Figure 1 and the detailed scheme is shown in Figure S1. The nanobody-based LFA employs a heterologous sandwich immunoassay involving two anti-ZIKV nanobodies, ZIKV_NbD6 and ZIKV_Nb32.^4^ The detection nanobody ZIKV_NbD6 is conjugated with AuNP and applied to the conjugate pad. ZIKV_Nb32 serves as the capture nanobody and is immobilized on the nitrocellulose (NC) membrane. In the presence of the ZIKV NS1 protein antigen, the AuNP-NbD6:NS1 complex is immobilized by ZIKV_Nb32 at the test line, resulting in a visible red band. Residual conjugated ZIKV_NbD6 is captured by an anti-VHH antibody at the control line, producing a second band. In the absence of the antigen, only the control line will display a band. The absence of a band at the control line indicates an invalid result (Figure 1).

### Nanobody Expression and Binding Characterization

**Figure 2:**
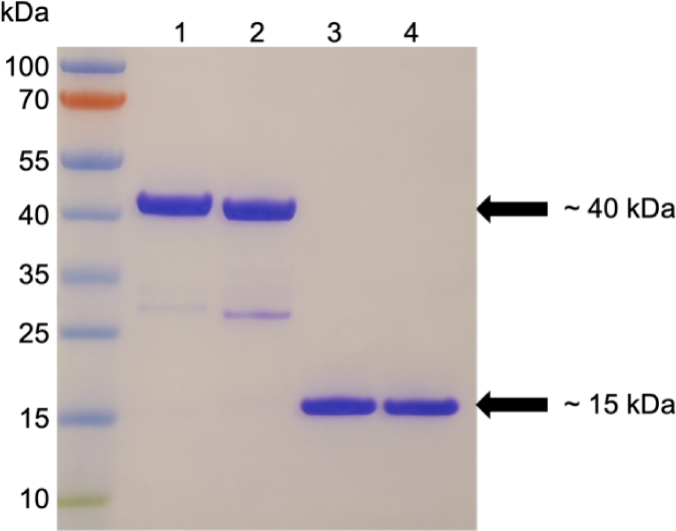
SDS-PAGE of purified nanobodies. 1: GST-NbD6 (39.7 kDa); 2: GST-Nb32 (39.7 kDa); 3: NbD6-Strep (14.9 kDa); 4: Nb32-Strep (15.1 kDa).

We evaluated two different expression and purification strategies for each of the two previously reported anti-ZIKV nanobodies: (i) cytoplasmic expression in *E. coli* T7 SHuffle using a GST-tag for solubilization and affinity purification, and (ii) periplasmic expression from *E. coli* BL21 (DE3) based on the pelB secretion signal and the much smaller StrepTag II affinity tag (Figure 2).

Both methods are assumed to foster the formation of the disulfide bridge that is part of the nanobody core structure.^43^ However, yields per liter of bacterial culture were generally low and did not indicate a clear preference for one or the other strategy (Table 2).

**Table 2:** Nanobody Expression Yields

Nanobody	Yield (mg per L of culture)
GST-Nb32	0.60
GST-NbD6	0.70
Nb32-Strep	1.50
NbD6-Strep	0.07

Periplasmic nanobody production is typically in the low mg range and usually lower than cytoplasmic expression.^44^ Nb32-Strep fell within this expected range (Table 2) while NbD6-Strep yield was very low. Yields were also relatively low for the expression of the GST-tagged nanobody versions from the *E. coli* SHuffle cytosol, even though we have recently used the identical protocol for high yield expression (up to 12 mg/L) of His-tagged anti-SARS-CoV-2 and anti-MERS nanobodies.^45^ This suggests that Nb32 and NbD6 may be more demanding expression targets and may require further optimizations for production scale up.

**Figure 3:**
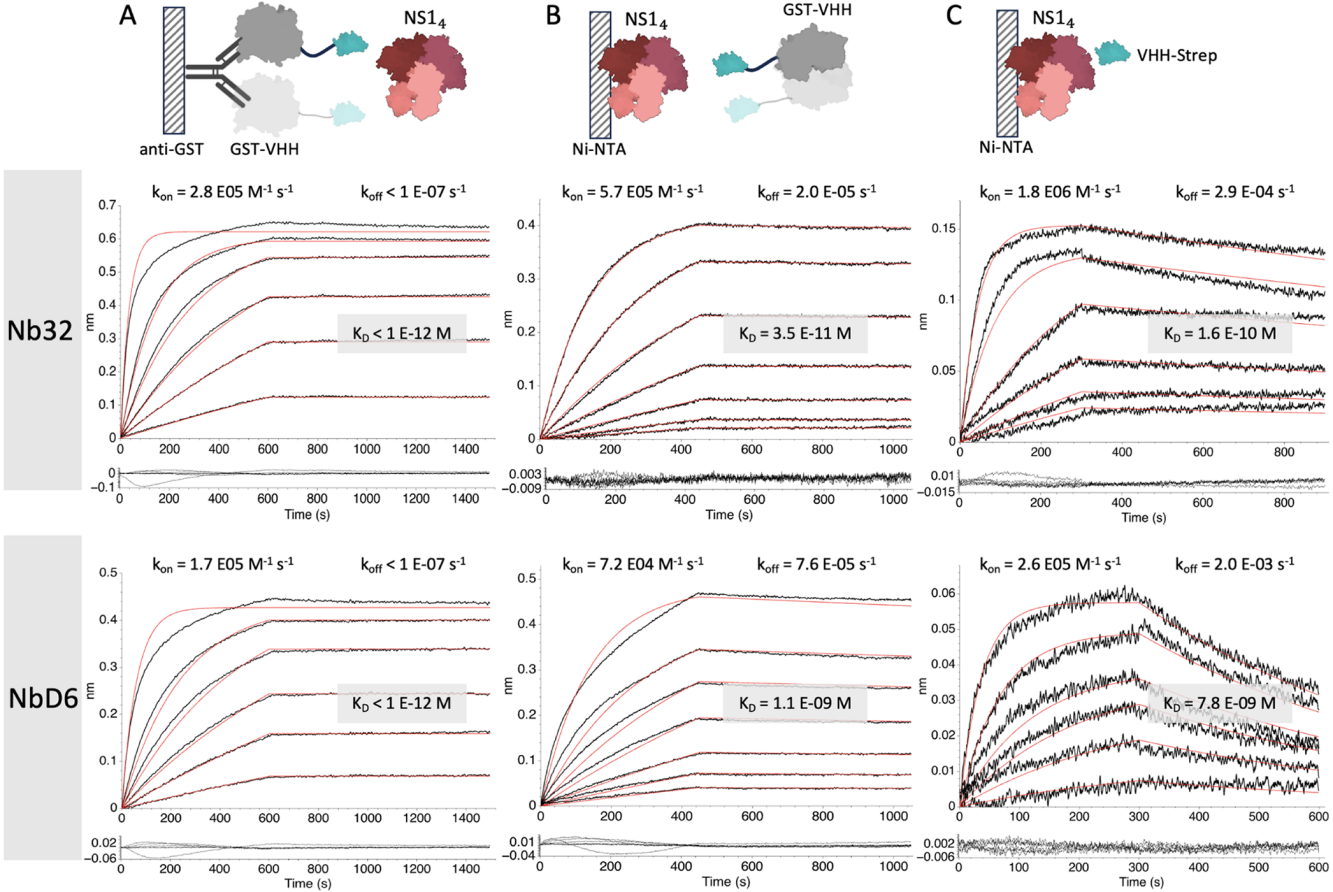
Binding analysis of ZIKV_Nb32 and ZIKV_NbD6 to the Zika NS1 antigen using BLI. Data were fit with a 1:1 binding model. (A) GST-tagged nanobodies immobilized on anti-GST sensors were exposed to a 2-fold dilution series of Zika NS1 starting at 100 nM. (B) NS1 target immobilized on Ni-NTA sensors exposed to the same GST-tagged nanobodies provided in the solution. The 2-fold dilution series started from 100 nM (Nb32) or 12.5 nM (NbD6). (C) Ni-NTA-immobilized NS1 exposed to StrepTag-II versions of both nanobodies using the same dilutions as in (B). The 13 nM trace was excluded as an outlier from the analysis of Nb32 (not shown). Structures of nanobodies are based on AlphaFold3 models.^46^ The NS1 tetramer and GST structures were taken from PDB (8WBG and 1GNW, respectively).

The original report of Nb32 and NbD6 only provided relative affinities estimated by ELISA.^4^ We therefore set out to quantify binding kinetics for both proteins by biolayer interferometry (BLI). We started with a setup comparable to the planned LFA setup whereby we were exposing nanobodies immobilized on sensor tips to the target protein in solution. The immobilization of GST-tagged nanobodies led to a surprisingly tight NS1 binding with unmeasurably low off rate resulting in an (unrealistically) low *K*_D_ below 1 pM (Figure 3A).

**Figure 4:**
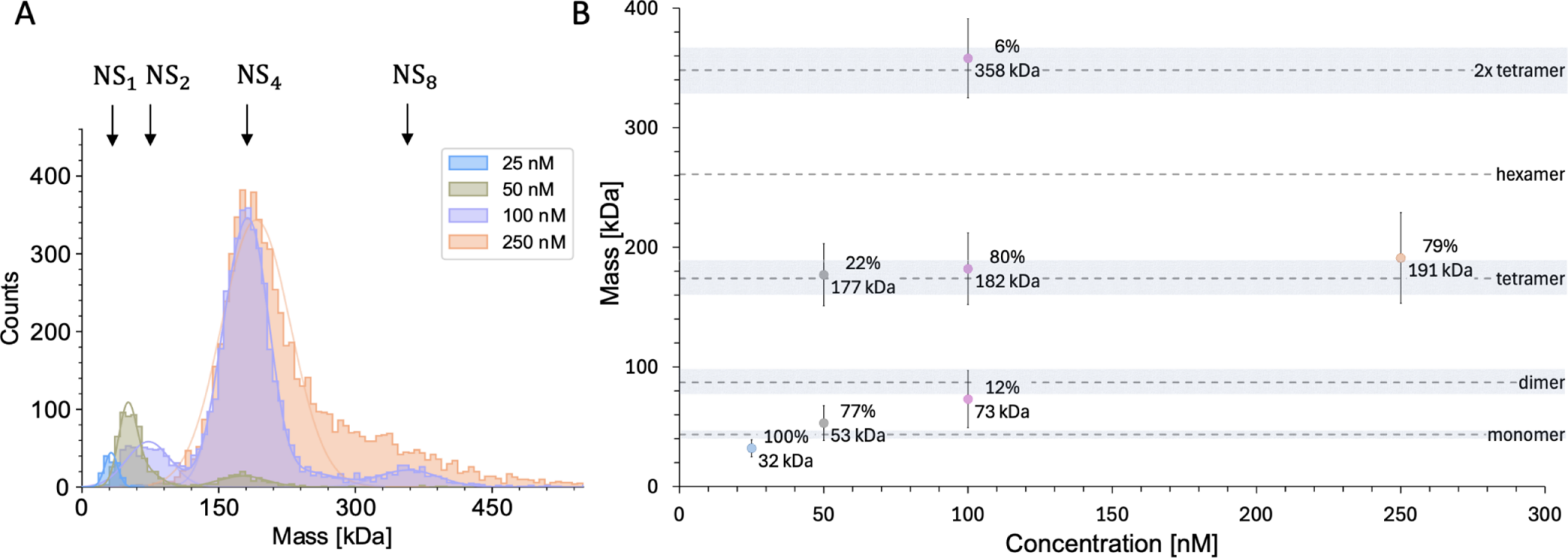
Characterization of Zika NS1 by mass photometry. (A) The contrast distribution of Zika NS1 single molecule collisions at varying concentrations (25, 50, 100, and 250 nM) indicated the presence of oligomeric species at higher concentrations. (B) Apparent molecular weights (±1 SD) deduced from contrast distribution peaks in A are plotted against NS1 (monomer) concentration (*M*_r_ of monomer = 43.5 kDa). The expected *M*_r_ for different oligomers (monomer *M*_r_ = 43.5 kDa) are shown as broken lines with shading indicating the average standard deviation (expected accuracy) from contrast distribution peaks in that size range.

We noted deviations from the 1:1 binding model (Figure S2) and wondered whether NS1 oligomerization might be contributing to binding artifacts. Various oligomer states have been observed for flavivirus NS1 glycoproteins.^4,47,48^ Dengue NS1 adopts a hexamer state in blood.^49^ and a mixture of oligomeric forms, including tetramers and hexamers, has been reported *in vitro*.^50^ By contrast, a tetrameric structure was proposed for the Zika NS1^50^ and a recent cryo-EM study described two different tetrameric (dimer of dimers) conformations.^51^ We examined our Zika NS1 target protein by mass photometry which estimates molecular mass from light scattering signals of single particle collisions with a glass surface (Figures 4A and S3). We observed a dynamic mixture of oligomeric states across the 25 to 100 nM concentration range that is accessible to the method. The monomer was prevalent at or below 50 nM whereas, in line with the previous report, the tetramer state dominated at 100 nM and above. However, we also found indications of dimers and dimers of tetramers (Figure 4B).

We thus considered it likely that NS1 avidity, i.e., the simultaneous binding of a single NS1 protein multimer to multiple immobilized nanobodies, could lead to an overestimation of affinity. In addition, individually captured NS1 monomers could also oligomerize on the surface preventing their unbinding. Indeed, the reverse orientation, with His-tagged NS1 immobilized and GST-tagged Nanobodies in solution, increased *k*_off_ by at least 2 orders of magnitude (Figure 3B). The result was a still very tight but now measurable equilibrium dissociation constant of 35 pM for Nb32 and 1.1 nM for NbD6. We wondered whether potential GST homodimerization among NS1-bound nanobodies was still creating avidity effects which would artificially reduce the off rate. We therefore repeated this experiment with StrepTag-II nanobodies lacking the GST domain (Figure 3C). This further multiplied the unbinding rate by a factor of 15-fold (Nb32) or 26-fold (Nb6) suggesting that GST dimerization had indeed reduced unbinding in the previous experiment. Interestingly, also the on-rate was (moderately) accelerated by 3.2-fold (Nb32) or 3.6-fold (Nb6), likely owing to the faster diffusion of the 2.6-fold lighter nanobody-only protein (15 kDa versus 40 kDa). The combined effects resulted in a *K*_D_ of 0.16 nM for Nb32 and 8 nM for NbD6 which we consider the best estimate for the interaction of the single nanobody with a single NS1 binding site.

**Figure 5:**
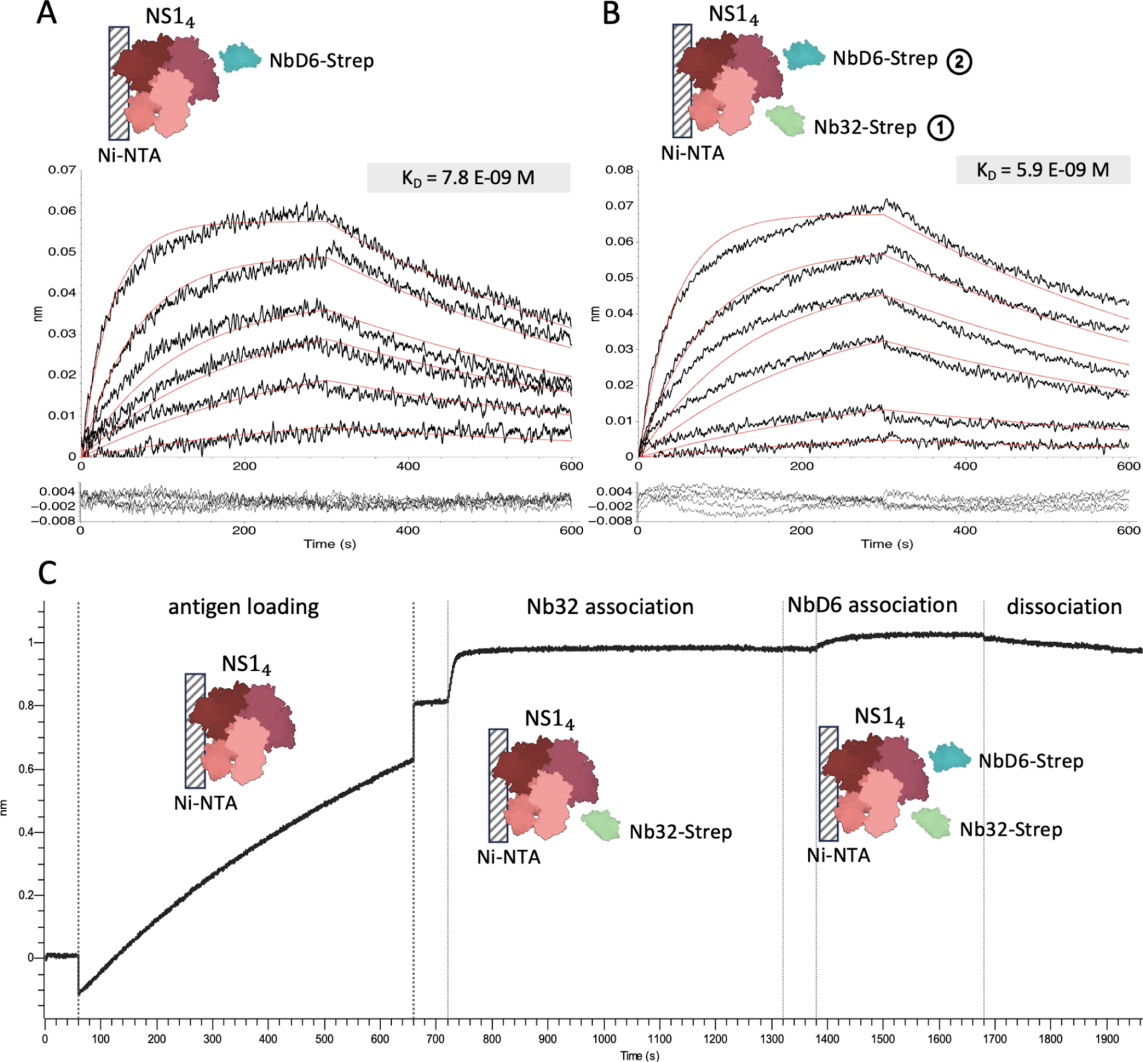
Competitive BLI assay for (A) binding of NbD6 alone (replicated from Figure 3C) and (B) binding of NbD6 after sensor saturation with Nb32. Results were fit to a 1:1 binding model. Zika NS1 was immobilized on Ni-NTA sensors as before. NbD6 was applied in varying concentrations (2-fold serial dilution starting from 100 nM). (C) representative sensor trace (50 nM NbD6) showing all the steps in the competitive assay. Structures of nanobodies are based on AlphaFold3 models.^46^ The NS1 tetramer and GST structures were taken from PDB (8WBG and 1GNW, respectively).

Our results confirm the tight interaction between both nanobodies and Zika NS1 antigen and indicate that the oligomeric state of NS1 should, in fact, further improve its capture on nanobody-coated surfaces. However, the precise structural arrangement remains unclear. We therefore sought to confirm that both nanobodies can bind simultaneously to distinct NS1 epitopes. Indeed, the initial binding of NbD32 did not significantly affect the subsequent binding kinetics of NbD6 (Figure 5). This supports the use of both nanobodies in a sandwich assay. Based on the stronger binding and slower dissociation, we chose to continue with Nb32 as the capture nanobody and NbD6 as the detection nanobody.

### Validation of the Nanobody-LFA

**Figure 6:**
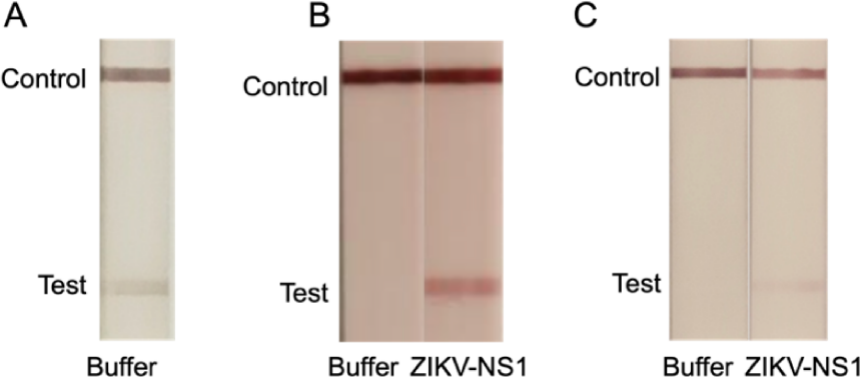
Validation of LFA using different nanobody constructs. NC membrane blocked with 0.6% BSA. (A) Capture Nb: GST-Nb32, detection Nb: GST-NbD6. (B) Capture Nb: Nb32-Strep, detection Nb: NbD6-Strep. (C) Capture Nb: GST-Nb32, detection Nb: NbD6-Strep.

Inspired by a previous report on the modular assembly of GST-tagged proteins onto gold nanoparticles,^52^ we initially built an LFA with the two GST-tagged nanobody versions. We printed NbD32-GST on the LFA nitrocellulose membrane and conjugated NbD6-GST with gold nanoparticles which were subsequently soaked into the LFA conjugate pad. Different concentrations of purified Zika NS1 were then added to the LFA sample pad and allowed to run for 15 min at room temperature. The resulting bands were visible to the naked eye (Figure 6). It should be noted that, when observed directly, the lines appear much clearer than in the photographs presented here. However, we observed a distinct band appearing at the test line even without the presence of antigen in the running buffer, indicating a false positive result (Figure 6A). We next assembled the LFA using only Strep-tagged nanobodies. As shown in Figure 6B, the buffer negative control sample displayed a distinct band only at the control line (printed with anti-VHH antibody). The NS1 sample showed both test and control lines indicating the specific binding between NbD6-Strep:AuNP, NS1 and immobilized NbD32.

To further explore the effect of the GST-tag, we also examined a mixed setup using a GST-tagged capture nanobody (NbD32-GST) alongside a Strep-tagged detection nanobody (NbD6-Strep:AuNP). This layout likewise eliminated the false positive results (Figure 6C). The initial false positive effect therefore likely stems from the dimerization between GST tags on both the detection and capture nanobodies.^53^ We therefore decided to assess sensitivities for both the StrepTag-only and the mixed StrepTag/GST-tag LFA architecture.

**Figure 7:**
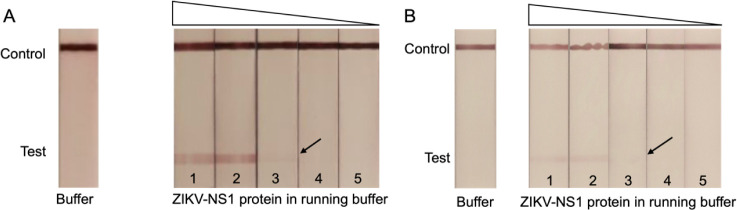
LOD determination of ZIKV LFA in running buffer. Lane1–7: dilution series of ZIKV NS1 protein. Lane 1: 2.5 μg/mL, lane 2: 250 ng/mL, lane 3: 25 ng/mL, lane 4: 2.5 ng/mL, lane 5: 0.25 ng/mL. (A) Capture Nb: Nb32-Strep, detection Nb: NbD6-Strep. NC membrane blocked with 0.6% BSA. (B) Capture Nb: GST-Nb32, detection Nb: NbD6-Strep. NC membrane blocked with 0.6% BSA.

We applied a 10-fold dilution series of ZIKV NS1 protein ranging from 2.5 μg/mL to 0.25 ng/mL to LFA test strips in order to determine the limit of detection (LOD) of both versions. Clear bands at the test line were visible at 25 ng/mL antigen concentration for both LFA versions (Figure 7). The LOD of the ZIKV nanobody-LFA falls within the typical range for such assays, and is comparable to the 18 ng/mL LOD reported in a previous monoclonal antibody-based sandwich LFA against ZIKV NS1 protein.^31^ We prioritized the Strep-tagged Nanobody LFA version for further experiments as it consistently yielded better visible bands with higher contrast.

### Serum and Urine Testing

**Figure 8:**
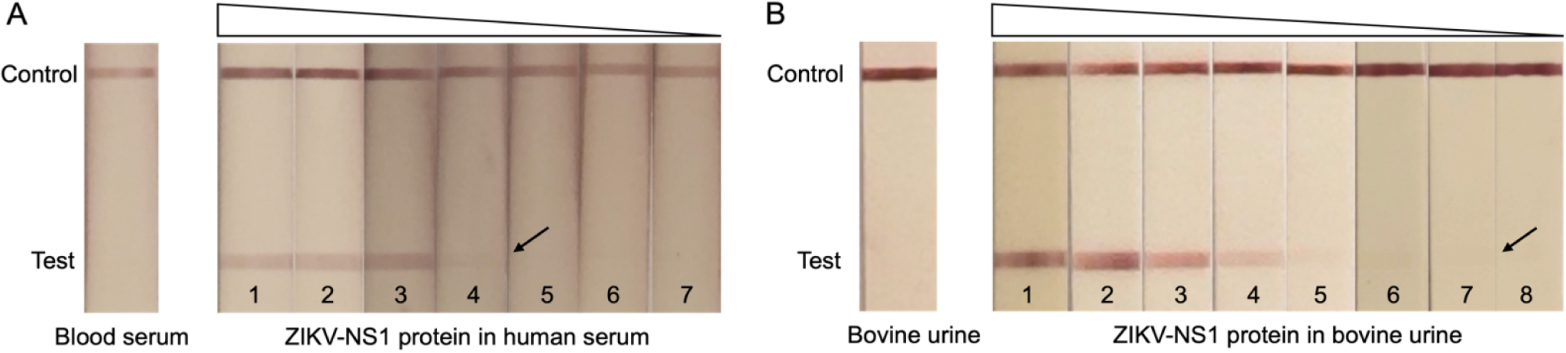
Validation of ZIKV LFA using Strep-tagged nanobodies in human serum (A) and bovine urine (B). Capture Nb: Nb32-Strep, detection Nb: NbD6-Strep. NC membrane blocked with 0.6% BSA. Lane1–8: dilution series of ZIKV NS1 protein. Lane 1: 2.5 μg/mL, lane 2: 0.5 μg/mL, lane 3: 0.1 μg/mL, lane 4: 20 ng/mL, lane 5: 4 ng/mL, lane 6: 2 ng/mL, lane 7: 1 ng/mL, lane 8: 0.5 ng/mL.

We next set out to test the Nanobody-LFA with body fluid samples. Serum and urine are two commonly used body fluids for early stage ZIKV detection.^9,54^ Among these, urine is the more easily accessible, noninvasive specimen. Moreover, urine has been reported to be more sensitive for Zika diagnosis than serum, with a longer detection duration.^54−57^ However, ZIKV NS1 levels in body fluids are still largely unknown.^58^ In a previous study using ZIKV-infected Vero cells, the extracellular concentration of ZIKV NS1 was found to be around 800 ng/mL.^31^ Using a human cell line, secreted ZIKV NS1 levels were observed to be within 400–500 ng/mL.^20^ Overall, high sensitivity is essential for ZIKV detection, and a suitable POC diagnostic should be in the nanogram detection range. To verify that the LFA can detect antigens not only in a defined running buffer but also in more complex physiological environments, we spiked different concentrations of recombinant NS1 protein into human serum and bovine urine. Despite the higher complexity of serum compared to running buffer, the test line did not show any false positive bands in the absence of antigen (Figure 8A). When the antigen was diluted in 100% human serum, a similar sensitivity to that in running buffer, 20 ng/mL, was observed (Figure 8A). This result is comparable to a previous aptamer-based ELISA analysis of ZIKV NS1 protein, which reached 10 ng/mL in 100% human serum.^18^ Somewhat surprisingly, the Nanobody-LFA performed better in bovine urine than both in buffer and human serum, achieving a 20-fold lower LOD of about 1 ng/mL NS1 protein (Figure 8B). We concluded that the nanobody-LFA detects the ZIKV NS1 protein in various physiological fluids at high sensitivity.

### Specificity

**Figure 9:**
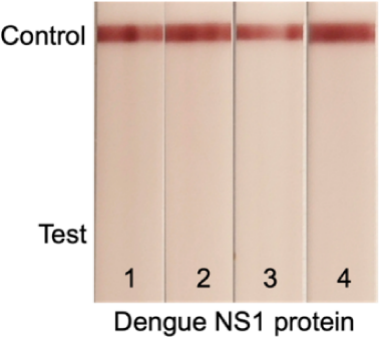
Dengue NS1 test in our LFA using Strep-tagged nanobodies. Capture Nb: Nb32-Strep, detection Nb: NbD6-Strep. NC membrane blocked with 0.6% BSA. Sample 1: 8.5 μg/mL Dengue serotype 1 NS1 protein, sample 2: 26.7 μg/mL Dengue serotype 2 NS1 protein, sample 3: 15.6 μg/mL Dengue serotype 3 NS1 protein, sample 4: 11.0 μg/mL Dengue serotype 4 NS1 protein.

One challenge of ZIKV diagnosis is the potential cross-reactivity with other flaviviruses, particularly with Dengue virus.^59−61^ The concentration of Dengue NS1 protein in patient serum is typically around 15 μg/mL 2 days postinfection.^58^ ZIKV NS1 concentrations are not yet well established but expected to be lower.^58^ We performed BLI binding assays between the recombinant NS1 proteins from all four Dengue serotypes and the two Zika nanobodies (Figure S4). In line with the ELISA results from the original nanobody study,^4^ we did not observe any cross-reactivity between Zika nanobodies and Dengue NS1 proteins. We then challenged our Nanobody-LFA with the four serotypes of Dengue NS1 at concentrations ranging from 8.5 μg/mL to 26.6 μg/mL. No positive bands were observed (Figure 9), demonstrating the high specificity of our LFA for ZIKV detection.

### Stability

**Figure 10:**
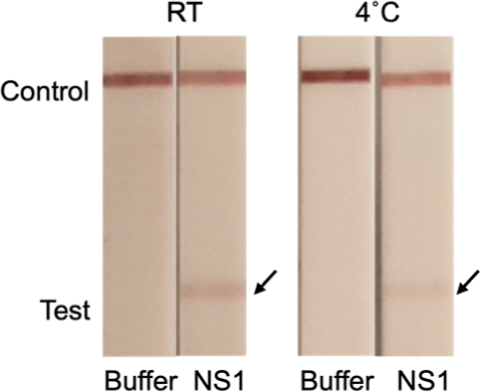
Stability test of our LFA using Strep-tagged nanobodies at room temperature or 4 °C. Capture Nb: Nb32-Strep, detection Nb: NbD6-Strep. NC membrane blocked with 0.6% BSA.

Storage stability is another critical feature for guaranteeing LFA affordability and accessibility. We tested the functionality of our LFA after one month of storage at both room temperature and at 4 °C. The lateral flow strips stored under both room temperature and refrigerated conditions were subjected to weekly functionality tests (Figure S5). All strips consistently demonstrated full functionality after storage for 28 days (Figure 10). However, the bands were not as clearly visible when stored at room temperature. This indicates that, while our LFA retains functionality over time, stability needs to be optimized for future applications. Possible approaches include the freeze-drying of decorated AuNPs or test strips in combination with buffer optimization and cryoprotectants.^62,63^ Nanobodies can typically be lyophilized^64^ with some studies reporting that cryoprotectants are unnecessary.^65,66^

## Conclusion and Outlook

ZIKV infections have been a concern for several decades, yet low-cost rapid detection methods remain elusive.^67^ We here developed a fully nanobody-based LFA for the detection of ZIKV NS1. Two previously developed anti-NS1 nanobodies recognize NS1 with very high affinity and high specificity. In particular, the lack of cross-reactivity with Dengue NS1 bodes well for real-world diagnostic applications. Interestingly, the oligomerization of the NS1 target protein appears to stabilize its capture on densely coated nanobody surfaces. We expect that such avidity-supported capture could also be utilized as a generally applicable design strategy for other multimeric targets. Among different purification strategies and purification tags, periplasmic Strep-tagged nanobodies achieved the highest detection sensitivity. However, protein yields need to be optimized. We prioritized oxidative expression conditions but did not explore whether the formation of the internal disulfide bond is in fact functionally important.

Compared to other established ZIKV detection methods, such as nucleic acid amplification, ELISA, and surface-enhanced Raman spectroscopy immunoassay,^68−70^ the nanobody-LFA offers the advantage of supporting rapid point of care or self-testing. The test is not only cheaper to manufacture but also easier to use, while still achieving relatively high sensitivity. Notably, we observed the highest sensitivity when the NS1 protein was detected from urine. Urine is a well-established medium for nucleic acid and antibody tests, and there is growing evidence that viral antigens can be carried over or even enriched in this easily accessible fluid.^71,72^ In fact, LFA-based detection of Zika antigens in urine could be compatible with standard pregnancy tests, suggesting the value of a combined test that could flag pregnancies at risk of Zika-related microcephaly. The ZIKV nanobody-LFA meets the World Health Organization’s ASSURED guidelines, which emphasize low cost, the ability to detect from unprocessed samples, and results that can be read with the naked eye.

## Data Availability

To aid in method adoption, practice, and troubleshooting, plasmids have been deposited to the Addgene repository (pET29b_GST-Nb32:228895, pET29b_GST-NbD6:228896, pJEx411c_pelB-NbD6-Strep2:228897, pJEx411c_pelB-N32-Strep2:228898).

## Supporting Information Available

Results of lateral flow strip scheme, binding measurements, stability test and nanobody and protein sequences (PDF)
