# Supplementary material for: Nanobody-Based Lateral Flow Assay for Rapid Zika Virus Detection
Source: ACS Synth Biol. 2025 Mar 7;14(3):890–900. doi: 10.1021/acssynbio.4c00819 (PMC11934133; doi:10.1021/acssynbio.4c00819)
Supplement: Supplementary file 1 — sb4c00819_si_001.pdf [file sb4c00819_si_001.pdf]

Supporting Information

Nanobody-Based Lateral Flow Assay for Rapid Zika Virus Detection

Yuli Peng[1,§], Atheer Alqatari[2,§], Fabian Kiessling[3], Dominik Renn[1,*], Raik Grünberg[2,*], Stefan T. Arold[2,*], Magnus Rueping[1,3,*]

[1] KAUST Catalysis Center (KCC), Division of Physical Sciences & Engineering, King Abdullah University of Science and Technology, KAUST, 23955 Thuwal, Kingdom of Saudi Arabia
[2] KAUST Center of Excellence for Smart Health, Biological and Environmental Science and Engineering Division, King Abdullah University of Science and Technology (KAUST), Thuwal 23955-6900, Kingdom of Saudi Arabia
[3]Institute for Experimental Molecular Imaging (ExMI), University Clinic, RWTH Aachen, Forckenbeckstraße 55, D-52074 Aachen, Germany

[§]These authors contributed equally.

***Correspondence:** dominik.renn@kaust.edu.sa, raik.grunberg@kaust.edu.sa, stefan.arold@kaust.edu.sa, magnus.rueping@kaust.edu.sa

Table of Content

Lateral Flow Strip Scheme	S3
BLI ZIKV NS1 binding analysis	S4
Calibration for mass photometry	S5
BLI Dengue NS1 binding curves	S6
Stability test	S7
Nanobody sequences	S8
NS1 protein sequence	S9

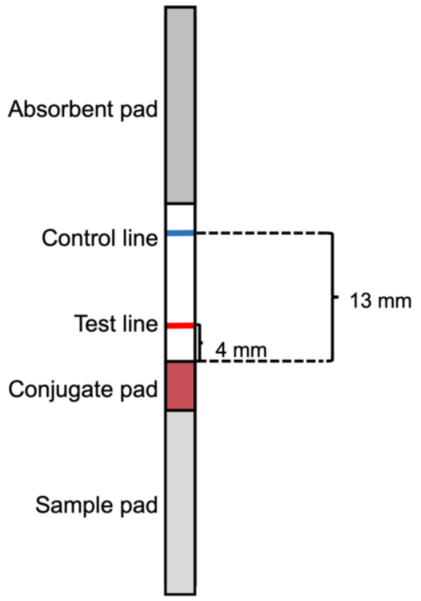

Figure S1. Detailed lateral flow strip scheme. Distances of test line and control line from conjugate pad are as indicated.

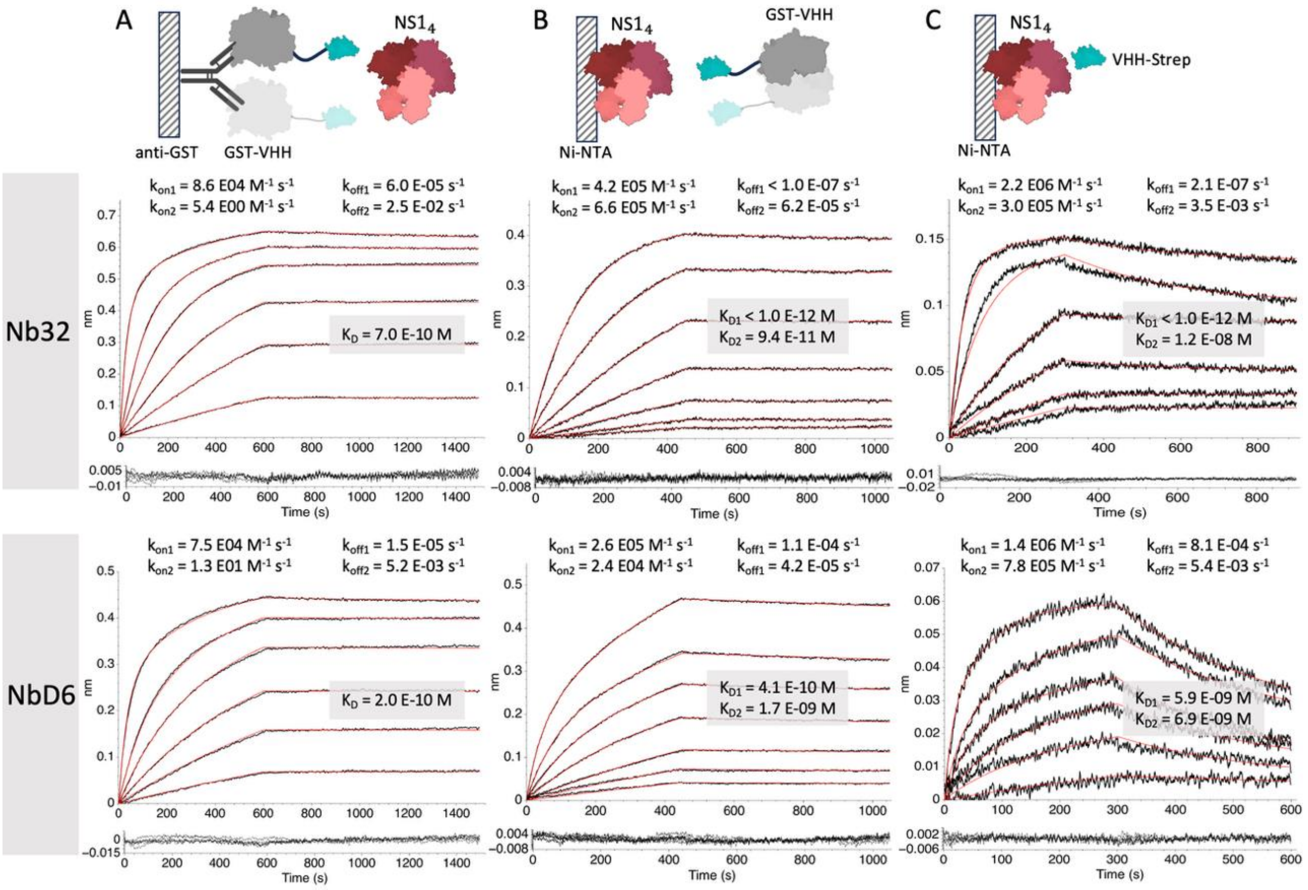

Figure S2. BLI binding analysis (replotted from Figure 2 using heterogenous and bivalent fit). (A) A bivalent 1:2 model fit was applied to the data, assuming analyte binding to two immobilized ligands. (B) and (C) A heterogeneous model fit was used, assuming the binding of two ligands to a single immobilized target at distinct sites. Structures of nanobodies are based on AlphaFold3 models. The NS1 tetramer and GST structures were taken from PDB (8WBG and 1GNW, respectively).

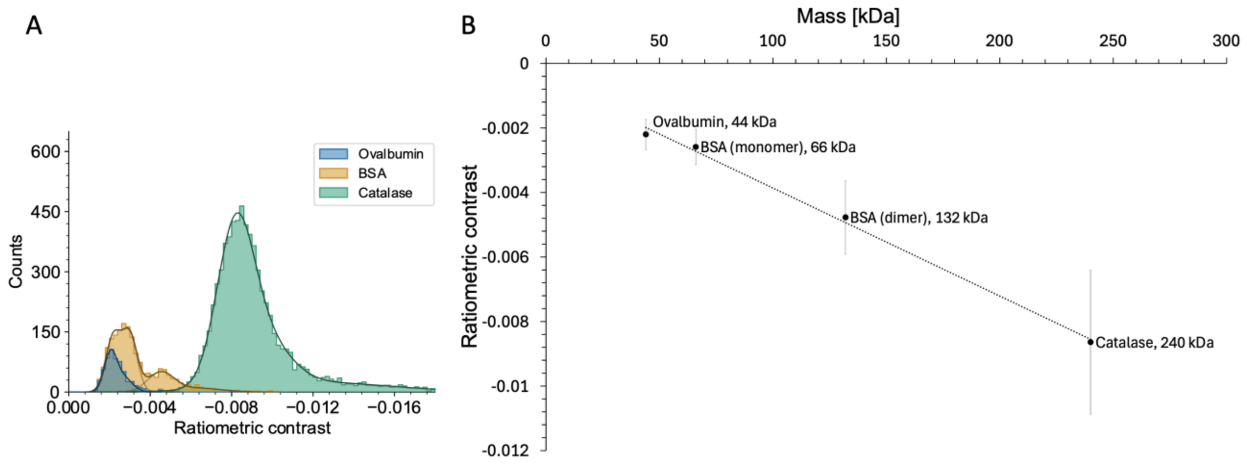

Figure S3. Calibration for mass photometry. (A) Contrast distribution of different protein standards - ovalbumin (50 nM), bovine serum albumin (BSA, 50 nM), and catalase (17 nM). (B) Calibration curve built from A. Error bars represent the standard deviation among collision events from the single measurement of each protein.

(A)

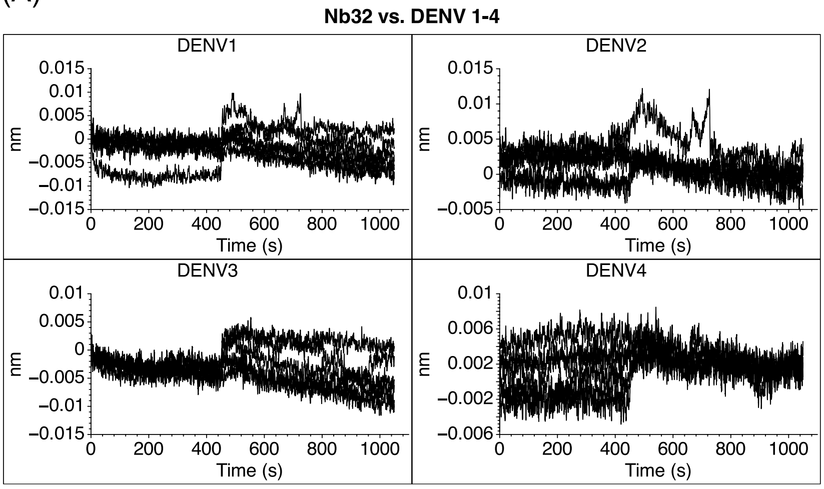

(B)

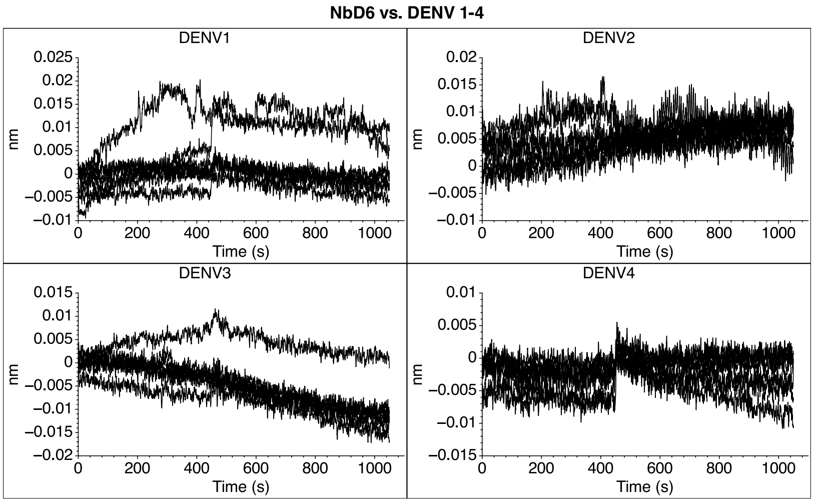

Figure S4. Nanobody *versus* Dengue NS1 binding curves using BLI. Cross-reactivity results for Nb32 (A) and NbD6 (B) against the four Dengue serotypes using BLI. The Dengue antigen was immobilized on Ni-NTA sensors at a constant concentration of 100 nM, while the nanobodies were provided in solution in a two-fold serial dilution starting at 100 nM. There is no indication for cross-reactivity (binding) at these concentrations.

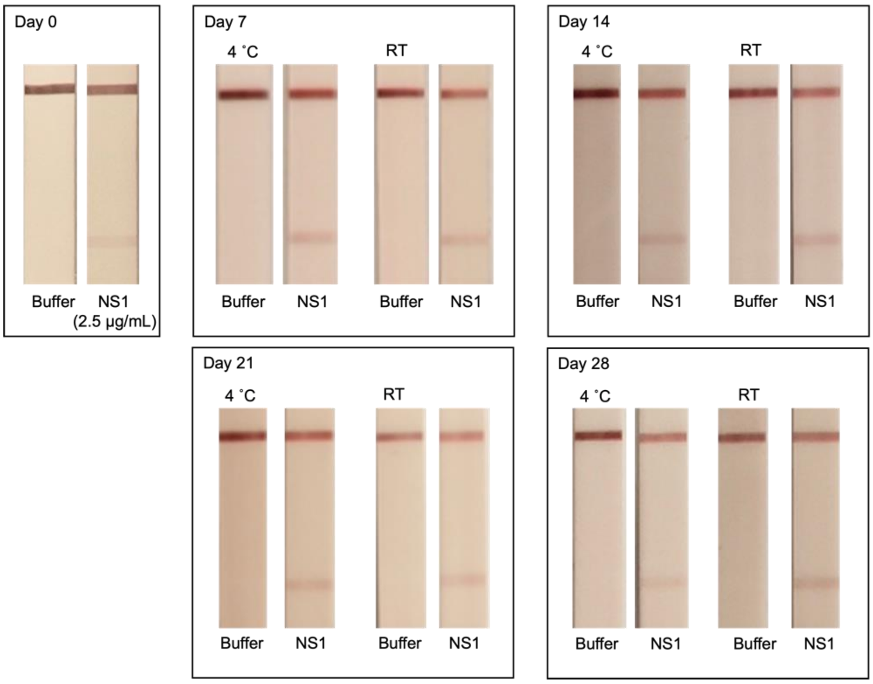

Figure S5. Stability test. Stability tests were conducted every 7 days. Test strips were stored at either 4°C or room temperature.

Supplementary 6. Nanobody sequences

NbD6-Strep<br>(pelB signal<br>peptide<br>underlined)	<u>KYLLPTAAAGLLLLAAQPAMA</u>EVQLVESGGGSVQPGGSLRLSCAASGF<br>AFSHYAMRWVRQAPGKGLEWVSVINSDGEDTWYADSVKGRFTISRD<br>NAKNTLYLQMNSLKPEDTGVYYCAIGRTHDTKGQGTQVTVSSSGLEVL<br>FQGPTGSAWSHPQFEKV
Nb32-Strep<br>(pelB signal<br>peptide<br>underlined)	<u>KYLLPTAAAGLLLLAAQPAMA</u>EVQLVESGGGLVQAGGSLRLSCAVSGID<br>FSRYAITWNRQSPGNQRREWVATLPPADTTVYADAVKGRFTISRDNTK<br>NTVYLQMNSLKPEDTAVYYCATSPRIHNWGQGTQVTVSSSGLEVLFQ<br>GPTGSAWSHPQFEKV
GST-NbD6	MSPILGYWKIKGLVQPTRLLLEYLEEKYEEHLYERDEGDKWRNKKFEL<br>GLEFPNLPYYIDGDVKLTQSMAIIRYIADKHNMLGGCPKERAEISMLEG<br>AVLDIRYGVSRIAYSKDFETLKVDFLSKLPEMLKMFEDRLCHKTYLNGD<br>HVTHPDFMLYDALDVVLYMDPMCLDAFPKLVCFKKRIEAIPQIDKYLKS<br>SKYIAWPLQGWQATFGGGDHPPKSDLVPRGSSSGSGSGSGSGSTGE<br>VQLVESGGGSVQPGGSLRLSCAASGFAFSHYAMRWVRQAPGKGLEW<br>VSVINSDGEDTWYADSVKGRFTISRDNAKNTLYLQMNSLKPEDTGVYY<br>CAIGRTHDTKGQGTQVTVS
GST-Nb32	MSPILGYWKIKGLVQPTRLLLEYLEEKYEEHLYERDEGDKWRNKKFEL<br>GLEFPNLPYYIDGDVKLTQSMAIIRYIADKHNMLGGCPKERAEISMLEG<br>AVLDIRYGVSRIAYSKDFETLKVDFLSKLPEMLKMFEDRLCHKTYLNGD<br>HVTHPDFMLYDALDVVLYMDPMCLDAFPKLVCFKKRIEAIPQIDKYLKS<br>SKYIAWPLQGWQATFGGGDHPPKSDLVPRGSSGSGSGSGSGSTGEV<br>QLVESGGGLVQAGGSLRLSCAVSGIDFSRYAITWNRQSPGNQRREWV<br>ATLPPADTTVYADAVKGRFTISRDNTKNTVYLQMNSLKPEDTAVYYCAT<br>SPRIHNWGQGTQVTVS

Supplementary 7. NS1 Protein sequence

ZIKV (SPH2015)	VGCSVDFSKKETRCGTGVFVYNDVEAWRDRYKYHPDSPRRLAAAVK<br>QAWEDGICGISSVSRMENIMWRSVEGELNAILEENGVQLTVVVGSVKN<br>PMWRGPQRLPVPVNELPHGWKAWGKSHFVRAAKTNNSFVVDGDTLK<br>ECPLKHRAWNSFLVEDHGFGVFHTSVWLKVREDYSLECDPAVIGTAVK<br>GKEAVHSDLGYWIESEKNDTWRLKRAHLIEMKTCEWPKSHTLWTDGIE<br>ESDLIIPKSLAGPLSHHNTREGYRTQMKGPWHSEELEIRFEECPGTKV<br>HVEETCGTRGPSLRSTTASGRVIEEWCCRECTMPPLSFRAKDGCWYG<br>MEIRPRKEPESNLVRSMVTAGSTDHMDHFSL
